# The National COVID‐19 Clinical Evidence Taskforce: pregnancy and perinatal guidelines

**DOI:** 10.5694/mja2.51729

**Published:** 2022-10-02

**Authors:** Caroline SE Homer, Vijay Roach, Leila Cusack, Michelle L Giles, Clare Whitehead, Wendy Burton, Teena Downton, Glenda Gleeson, Adrienne Gordon, Karen Hose, Jenny Hunt, Jackie Kitschke, Nolan McDonnell, Philippa Middleton, Jeremy JN Oats, Antonia W Shand, Kellie Wilton, Joshua Vogel, Julian Elliott, Steven McGloughlin, Steve J McDonald, Heath White, Saskia Cheyne, Tari Turner, for the National COVID‐19 Clinical Evidence Taskforce

**Affiliations:** ^1^ Maternal, Child and Adolescent Health Program Burnet Institute Melbourne VIC; ^2^ Royal North Shore Hospital Sydney NSW; ^3^ Mater Hospital Sydney NSW; ^4^ Cochrane Australia Monash University Melbourne VIC; ^5^ Monash University Melbourne VIC; ^6^ Monash Health Melbourne VIC; ^7^ Royal Women’s Hospital Melbourne VIC; ^8^ University of Melbourne Melbourne VIC; ^9^ Morningside General Practice Clinic Brisbane QLD; ^10^ Australian College of Rural and Remote Medicine Brisbane QLD; ^11^ Central Australia Health Service Alice Springs NT; ^12^ CRANAplus Adelaide SA; ^13^ Sydney Local Health District Sydney NSW; ^14^ Sydney Institute for Women Children and their Families Sydney NSW; ^15^ Royal Brisbane Hospital Brisbane QLD; ^16^ Victorian Aboriginal Health Service Melbourne VIC; ^17^ Women's and Children's Hospital Adelaide SA; ^18^ King Edward Memorial Hospital Perth WA; ^19^ University of Western Australia Perth WA; ^20^ SAHMRI, Women and Children's Hospital Adelaide SA; ^21^ University of Adelaide Adelaide SA; ^22^ Royal Hospital for Women Sydney NSW; ^23^ University of NSW Sydney NSW; ^24^ Australian College of Midwives Canberra ACT; ^25^ NHMRC Clinical Trials Centre University of Sydney Sydney NSW

**Keywords:** COVID‐19, Maternal health, Fetomaternal medicine, Neonatology

## Abstract

**Introduction:**

Pregnant women are at higher risk of severe illness from coronavirus disease 2019 (COVID‐19) than non‐pregnant women of a similar age. Early in the COVID‐19 pandemic, it was clear that evidenced‐based guidance was needed, and that it would need to be updated rapidly. The National COVID‐19 Clinical Evidence Taskforce provided a resource to guide care for people with COVID‐19, including during pregnancy. Care for pregnant and breastfeeding women and their babies was included as a priority when the Taskforce was set up, with a Pregnancy and Perinatal Care Panel convened to guide clinical practice.

**Main recommendations:**

As of May 2022, the Taskforce has made seven specific recommendations on care for pregnant women and those who have recently given birth. This includes supporting usual practices for the mode of birth, umbilical cord clamping, skin‐to‐skin contact, breastfeeding, rooming‐in, and using antenatal corticosteroids and magnesium sulfate as clinically indicated. There are 11 recommendations for COVID‐19‐specific treatments, including conditional recommendations for using remdesivir, tocilizumab and sotrovimab. Finally, there are recommendations not to use several disease‐modifying treatments for the treatment of COVID‐19, including hydroxychloroquine and ivermectin. The recommendations are continually updated to reflect new evidence, and the most up‐to‐date guidance is available online (https://covid19evidence.net.au).

**Changes in management resulting from the guidelines:**

The National COVID‐19 Clinical Evidence Taskforce has been a critical component of the infrastructure to support Australian maternity care providers during the COVID‐19 pandemic. The Taskforce has shown that a rapid living guidelines approach is feasible and acceptable.

The coronavirus disease 2019 (COVID‐19) pandemic has created considerable challenges for the delivery of health care globally. A novel coronavirus meant a lack of knowledge and understanding on the nature of the infection, including a lack of data on epidemiology, transmission mechanisms, progression of the disease, and treatment options for people infected with severe acute respiratory syndrome coronavirus 2 (SARS‐CoV‐2). Much of the initial clinical advice relied on extrapolation of data and experience with other respiratory viruses. However, there was an understanding that the behaviour of SARS‐CoV‐2 required specific understanding of the pathophysiology and approach to medical management. Very quickly, by early 2020, considerable research started to be published as clinicians in most countries were challenged with how to care for people with COVID‐19. There has been a substantial surge in publications relating to COVID‐19.^1^ By the end of April 2022, it was estimated that more than 1 million articles on COVID‐19 had been published,^2^ posing an important challenge for clinicians, health service planners and administrators who need to stay abreast of new developments.

This unprecedented volume of COVID‐19 research and the pace at which it has been emerging^3^ highlight the need for rapid and continually updated evidence synthesis to ensure that health decision making is based on current evidence. Living guidelines are based on systematic reviews that are continually updated as new research becomes available, ensuring that decisions are made on the currently available evidence.^4^

## National COVID‐19 Clinical Evidence Taskforce

Recognising the pressing need for reliable and up‐to‐date COVID‐19 clinical care recommendations for frontline health care providers in Australia, the National COVID‐19 Clinical Evidence Taskforce was established in March 2020 to develop living, evidence‐informed guidelines for primary, hospital and critical care of people with COVID‐19.^5^ A living approach to guidelines provides timely advice for decision makers by optimising the development process: it involves the prioritisation of areas where guidance is needed, continual surveillance for emerging evidence, and incorporation of new research evidence into guideline recommendations.^6^ This approach is particularly pertinent in scenarios, such as the COVID‐19 pandemic, where evidence is emerging rapidly, current evidence is unclear, and new research might affect health policy or practice decisions.^4^

The Taskforce is a member of the Australian Living Evidence Consortium, based at Cochrane Australia, which is pioneering several living guideline projects and methodology research. The Taskforce comprises 34 peak Australian health professional bodies whose members provide clinical care for people with COVID‐19. In the context of maternal and newborn health, these include: the Australian College of Midwives, the Royal Australian and New Zealand College of Obstetricians and Gynaecologists, the Royal Australasian College of Physicians, the Australian and New Zealand College of Anaesthetists, the Australian College of Neonatal Nurses, CRANAplus (professional body for the remote health workforce), the Royal Australian College of General Practitioners and the Australian College of Rural and Remote Medicine.^5^ A complete list of the Taskforce’s member organisations can be found on the Taskforce website.^7^

Through continual surveillance of the available evidence, systematic reviewers and guideline methodologists from within the Taskforce identify, evaluate and synthesise new research to ensure the evidence that underpins the guideline recommendations is up to date. The evidence is reviewed by panels of clinical experts from across Australia, who follow rigorous and transparent methods based on the Grading of Recommendations Assessment, Development and Evaluation (GRADE) approach to developing evidence‐based guidelines.^5^

Consequently, the Taskforce recommendations are updated regularly, as new evidence emerges.^5^ To facilitate the translation of new and updated guideline recommendations into clinical practice, the Taskforce maintains a series of clinical flowcharts for point‐of‐care use.^8^ While the Taskforce is supported by several organisations, as well as state and federal governments, it remains independent — its funders play no role in the development of the guidelines. Conflicts of interest for all participating panel members are assessed before their involvement in decision‐making processes.^5^

## COVID‐19 during pregnancy and perinatal care

Although the focus of many of the COVID‐19 publications and the initial guidance was care for people with acute SARS‐CoV‐2 infection, especially in the critical or intensive care context, every area of health care was affected. This included pregnancy and perinatal care, and studies with a focus on pregnant and breastfeeding women emerged rapidly. Early studies expressed concerns about the safety of vaginal birth and breastfeeding in women with COVID‐19^9^, ^10^ and transmission of infection to the baby.^11^, ^12^ An initial report recommended that partners be excluded from the birth, assisted vaginal birth was preferred, and early cord clamping should be practised with no skin‐to‐skin contact after birth;^13^ another recommended separating the mother and baby.^14^ Early observational evidence indicated that pregnant and breastfeeding women were at greater risk than non‐pregnant women with regard to infection and adverse outcomes, including the need for hospitalisation, oxygen therapy and ventilation; they also showed that babies of mothers with COVID‐19 were at higher risk of stillbirth and pre‐term birth.^14^, ^15^, ^16^ This was especially so when the Delta variant predominated and during the period before widespread vaccination.^17^ Further challenges to caring for pregnant women with COVID‐19 included difficulties in using measures such as nursing in the prone position. Notably, pregnant women were largely excluded from COVID‐19‐related randomised clinical trials^17^ and, anecdotally, maternity service providers in Australia were uncertain as to the best practice to implement. It was on this background of uncertainty and challenges to clinical care that the Pregnancy and Perinatal Care Panel was established as part of the National COVID‐19 Clinical Evidence Taskforce.

The multidisciplinary Panel currently includes 14 experts, with diverse representation of setting, culture, geography and gender from across Australia, along with representation from urban, rural and remote areas.^18^ Specialties covered include Aboriginal and Torres Strait Islander health, general practice, infectious diseases, obstetrics and gynaecology, obstetric anaesthesia, midwifery, neonatology, perinatal epidemiology, and public health.^18^ The Panel considers research findings and develops recommendations aligned with Australian maternity care principles, which include the need for evidence‐based, person‐centred care that is responsive to the woman’s needs and preferences.^19^

## Methods

Taskforce partners collaborate to produce living guidelines that are developed according to the standards of the Australian National Health and Medical Research Council (NHMRC), which includes the use of GRADE methodology. The Evidence to Decision framework^20^ embedded within the MAGIC (Making GRADE the Irresistible Choice) digital platform (https://magicevidence.org) is used to publish the updated guideline recommendations. The recommendations published by the Taskforce are approved by the NHMRC on a regular basis. The living guideline methodology has been described in detail previously^5^ and is also available via the National COVID‐19 Clinical Evidence Taskforce website (https://covid19evidence.net.au/more‐about‐the‐guidelines).

To begin the process, clinical questions are identified by engaging with stakeholders and using surveillance strategies. The questions are then selected and prioritised for a living approach by the expert panels, based on:

- likely impact on patient outcomes;
- proportion of clinical population affected;
- extent of variation in current practice;
- likelihood of new evidence emerging; and
- uncertainty in the existing evidence base and strength of the recommendations.

Databases and other sources, such as preprint servers, are searched daily under the guidance of an information specialist and, where possible, relevant data are extracted from identified studies by the evidence team using a combination of Cochrane’s RevMan software^21^ and MAGIC. New evidence is presented to the relevant expert panel to develop guideline recommendations, which are then approved by the National Guidelines Leadership Group. Convened in collaboration with the Consumers Health Forum of Australia, a Consumer Panel provides further input on preferences and values relating to the guideline recommendations. The National Steering Committee, comprised of representatives from all the member organisations, provides oversight of the Taskforce and is responsible for endorsing the guideline recommendations. The living guidelines developed by the Taskforce are updated weekly or fortnightly, which is (to our knowledge) the shortest update cycle for any living guidelines.^5^

### Developing recommendations using GRADE methodology

Many factors are considered in developing recommendations. While GRADE is a systematic approach to rating the certainty of evidence, the GRADE process for developing recommendations involves further considerations. These include framing the health care question, selecting and rating the importance of the outcomes, summarising the evidence, assessing the certainty of the evidence, and converting the evidence into recommendations.^22^ Key factors that are considered when developing recommendations include benefits and harms, certainty of evidence, preferences and values of patients and other key stakeholders, resources, cost‐effectiveness considerations, feasibility, acceptability, and equity.^22^ The relevant GRADE assessments for each recommendation are presented within the online platform that is used to structure the guidelines (MAGICapp; https://magicevidence.org/magicapp/).

Recommendations are considered strong when most or all individuals will be best served by the recommended course of action, and they are considered conditional when not all individuals will be best served by the recommended course of action and there is a need to consider the individual patient’s circumstances, preferences and values. Strengths of recommendations used in the GRADE methodology are described in Box 1.

Box 1 **Strengths of recommendations used in the Grading of Recommendations Assessment, Development and Evaluation methodology^30^**

**Table:** 

**Strong recommendation**
Benefits outweigh harms for almost everyone. All or nearly all informed patients would likely want this option. This essentially means “just do it” and there is good reason to believe that all informed patients would want this option. The evidence should be of high or moderate quality, but in some instances strong recommendations can be issued based on low or very low quality evidence.
**Conditional (or weak) recommendation**
Benefits outweigh harms for the majority, but not for everyone. The majority of patients would likely want this option. A conditional recommendation does not necessarily mean that the guideline panel did not find sufficient evidence to support the suggested course of action. Indeed, there are two fundamental reasons for a conditional recommendation: the evidence is low quality, so it is hard to be sure of the right course of action, or there is a fine balance between benefits and harms of treatment alternatives. Other drivers for conditional recommendations are variability in patients’ values and preferences or issues relating to resource use, feasibility or equity. The implications of a conditional recommendation might be difficult to understand for clinicians without further explanation. In general, clinicians should think twice and consider individual patient factors when applying conditional recommendations. For example, patients’ values and preferences, comorbidities, polypharmacy, burden of medical care or personal limitations might result in the alternative course of action being the preferred option. Shared decision making would be mandated for most conditional recommendations.
**Consensus recommendation**
A consensus recommendation is used when there is not enough evidence to give an evidence‐based recommendation, but the relevant clinical panel still regards it as important to give a recommendation. A consensus recommendation can be given for or against an intervention and is based on the experience and expertise of the panel together with any available evidence.

Much of the available data on the management of COVID‐19 does not include pregnant women, so the expert panel often need to base their decisions on indirect evidence.

### Methods specific to pregnancy and perinatal care

The Pregnancy and Perinatal Care Panel meets regularly to provide guideline recommendations for population‐specific COVID‐19 care topics (eg, breastfeeding and umbilical cord clamping), and to review the living guideline recommendations for other populations to develop population‐specific guidance where appropriate. Although properly performed systematic reviews are the preferred source of evidence, for many of the clinical questions specific to pregnancy and perinatal care, direct trial evidence has usually not been available and thus lower certainty evidence (eg, observational studies) needs to be considered.^18^ In this regard, groups conducting living evidence synthesis, such as the COVID‐19 in Pregnancy initiative at the University of Birmingham,^23^ play a useful part in complementing the work of the Taskforce.

Guideline recommendations developed for adults by the Disease‐Modifying Treatment and Chemoprophylaxis Panel are subsequently reviewed by other panels, including the Pregnancy and Perinatal Care Panel and the Paediatric and Adolescent Care Panel. These panels in particular have met substantial challenges given that children and pregnant and breastfeeding women have regularly been excluded from COVID‐19 clinical trials.^24^, ^25^ Faced with limited data for the specific populations they represent, these panels consider the available evidence in non‐pregnant adults, including safety and pharmacokinetic data, to make judgements about intervention efficacy and effectiveness.^3^ Clinical experience from within the expert panel in using therapeutic agents in pregnancy for non‐COVID‐19 indications (eg, dexamethasone and budesonide) is also drawn on. The resulting recommendations acknowledge the limitations of the available evidence, including the risks of extrapolating, to offer much needed guidance despite the paucity of direct data. Adhering to GRADE methods,^26^ the certainty of the evidence is downgraded when the available studies do not include the relevant subpopulations. In cases where there are no trial data in the specific population, the panels produce consensus recommendations based on data from similar studies, best practice and expert consensus.^27^

## Recommendations

The Taskforce guidance for pregnant and breastfeeding women includes recommendations on the approach to care for women with COVID‐19, including mode of birth, delayed cord clamping, breastfeeding, skin‐to‐skin contact after birth and rooming‐in. These areas were prioritised at the outset owing to concerns about caring for women with COVID‐19 and the consequences of high levels of restrictive practice, and they are revisited regularly (Box 2). We did not prioritise the topic of whether partners should be excluded from the birth. The next priority was developing recommendations on therapies that pregnant and breastfeeding women with COVID‐19 could be offered (Box 3).

Box 2 Summary of recommendations on care for pregnant and breastfeeding women^*^

**Table:** 

Care approach	Strength of recommendation	Recommendation
Antenatal corticosteroids	Consensus recommendation	The use of antenatal corticosteroids for women at risk of pre‐term birth is supported as part of standard care, independent of the presence of COVID‐19.
Magnesium sulfate	Consensus recommendation	The use of magnesium sulfate in pregnancy for fetal neuroprotection for women at risk of pre‐term birth is supported as part of standard care, independent of the presence of COVID‐19.
Mode of birth	Conditional recommendation	For pregnant women with COVID‐19, mode of birth should remain as per usual care.
Delayed umbilical clamping	Consensus recommendation	Delayed umbilical cord clamping is supported as part of standard care, independent of the presence of COVID‐19.
Skin‐to‐skin contact	Consensus recommendation	Early skin‐to‐skin contact after birth and during the post‐natal period is supported, independent of the presence of COVID‐19. However, parents with COVID‐19 should use infection prevention and control measures (mask and hand hygiene).
Breastfeeding	Conditional recommendation	Breastfeeding is supported irrespective of the presence of COVID‐19. However, women with COVID‐19 who are breastfeeding should use infection prevention and control measures (mask and hand hygiene) while infectious.
Rooming‐in	Conditional recommendation	For women with COVID‐19 who have given birth, support rooming‐in of mother and newborn in the birth suite and on the post‐natal ward when both mother and baby are well. However, women with COVID‐19 should use infection prevention and control measures (mask and hand hygiene).

* Up to date as of 16 June 2022 (see https://covid19evidence.net.au/#living‐guidelines for the most recent recommendations). COVID‐19 = coronavirus disease 2019.

Box 3 Overview of therapy recommendations for pregnant and breastfeeding women^*^

**Table:** 

Therapy	Strength or type of recommendation	Recommendation
Casirivimab plus imdevimab	Conditional recommendation	Consider using casirivimab plus imdevimab within 7 days of symptom onset in pregnant or breastfeeding women with COVID‐19 who do not require supplemental oxygen and have one or more risk factors for disease progression.
Corticosteroids (inhaled)	Conditional recommendation	Consider using inhaled corticosteroids (budesonide or ciclesonide) within 14 days of symptom onset in adults with COVID‐19 who do not require supplemental oxygen and who have one or more risk factors for disease progression. Budesonide and ciclesonide are safe to use in pregnant and breastfeeding women.
Corticosteroids (systemic)	Recommended	Use dexamethasone 6 mg intravenously or orally for up to 10 days in pregnant or breastfeeding women with COVID‐19 who require supplemental oxygen (including mechanically ventilated patients).
Molnupiravir	Only in research settings	Do not use molnupiravir for the treatment of COVID‐19 in pregnant or breastfeeding women outside of randomised clinical trials with appropriate ethics approval.
Nirmatrelvir plus ritonavir	Only in research settings	Do not use nirmatrelvir plus ritonavir for the treatment of COVID‐19 in pregnant or breastfeeding women outside of randomised clinical trials with appropriate ethics approval.
Baricitinib	Only in research settings	Do not use baricitinib for the treatment of COVID‐19 in pregnant or breastfeeding women outside randomised clinical trials with appropriate ethics approval.
Sarilumab	Only in research settings	Do not use sarilumab for the treatment of COVID‐19 in pregnant or breastfeeding women outside randomised clinical trials with appropriate ethics approval.
Tocilizumab	Conditional recommendation	Consider using tocilizumab (see the National COVID‐19 Clinical Evidence Taskforce website for doses) for the treatment of COVID‐19 in pregnant or breastfeeding women who require supplemental oxygen, particularly where there is evidence of systemic inflammation.
Remdesivir	Conditional recommendation	Consider using remdesivir in pregnant or breastfeeding women hospitalised with COVID‐19 who require supplemental oxygen but do not require non‐invasive or invasive ventilation.
Sotrovimab	Conditional recommendation	Consider using sotrovimab within 5 days of symptom onset in pregnant women with COVID‐19 in the second or third trimester who do not require oxygen and who have one or more additional risk factors for disease progression (see the National COVID‐19 Clinical Evidence Taskforce website for risk factors).
Tixagevimab plus cilgavimab	Only in research settings	Do not use tixagevimab plus cilgavimab for the treatment of COVID‐19 in pregnant or breastfeeding women outside of randomised clinical trials with appropriate ethics approval.
VTE prophylaxis	Consensus recommendation	For pregnant or post‐partum women who are admitted to hospital (for any indication) and who have COVID‐19, use prophylactic doses of anticoagulants, preferably LMWH (eg, enoxaparin 40 mg once daily or dalteparin 5000 IU once daily) unless there is a contraindication, such as risk of major bleeding or imminent birth.
	Consensus recommendation	For pregnant or post‐partum women who are self‐isolating at home with mild COVID‐19 and where additional risk factors for VTE are present, consider using prophylactic doses of anticoagulants, preferably LMWH (eg, enoxaparin 40 mg once daily or dalteparin 5000 IU once daily) unless there is a contraindication, such as risk of major bleeding or imminent birth. Prophylactic anticoagulants should be continued for at least 14 days or until COVID‐19‐related morbidity (including immobility, dehydration and shortness of breath) has resolved.
	Consensus recommendation	For post‐partum women who have had COVID‐19 during pregnancy, consider using at least 14 days of prophylactic dosing of anticoagulants, preferably LMWH (eg, enoxaparin 40 mg once daily or dalteparin 5000 IU once daily) unless there is a contraindication, such as risk of major bleeding. Increased duration of 6 weeks should be considered if severe or critical COVID‐19 and/or additional risk factors for VTE are present.
Other disease‐modifying treatments that are not recommended	Not recommended	Do not use aspirin, azithromycin, colchicine, convalescent plasma, hydroxychloroquine, hydroxychloroquine plus azithromycin, interferon‐β‐1a, lopinavir–ritonavir, interferon‐β‐1a plus lopinavir–ritonavir or ivermectin for the treatment of COVID‐19.

* Up to date as of 24 April 2022 (see https://covid19evidence.net.au/#living‐guidelines for detailed recommendations). COVID‐19 = coronavirus disease 2019. LMWH = low‐molecular‐weight heparin. VTE = venous thromboembolism.

## Impact of the guidelines

The National COVID‐19 Clinical Evidence Taskforce has been a critical part of the architecture to support the Australian health system during the COVID‐19 pandemic. The Taskforce has shown that the rapid living guidelines approach is feasible and acceptable.^28^

Towards the end of 2020, a process evaluation was conducted, which targeted Australian health care practitioners who potentially provide care to individuals with suspected or confirmed COVID‐19. It showed that awareness of the work of the Taskforce was high and the vast majority of respondents reported that the guidelines were very or extremely relevant, easy to use, trustworthy and valuable.^29^ The evaluation highlighted that the guidelines had been a reliable, united source of evidence‐based advice, built confidence among health care practitioners in providing care, and provided them with reassurance in clinical decision making. The Taskforce website, flowcharts and recommendations have been widely used in Australia and overseas. As of 6 September 2022, the Taskforce website has been accessed by more than 625 000 users across 200 countries and territories, with over 1.2 million page views and more than 125 000 flowchart views.

## Conclusion

The pregnancy and perinatal guidelines produced by the National COVID‐19 Clinical Evidence Taskforce have been, and continue to be, an essential resource for doctors, nurses and midwives who provide acute and primary maternity care services. They are developed using highly trusted methods, and are endorsed by the NHMRC and 34 health professional organisations. The guideline development process is dynamic and rapid, which means that the guidance is updated quickly as new evidence is released. With thousands of papers being published each week on COVID‐19 epidemiology and treatments, the Taskforce has been a beacon of clarity for clinicians and consumers.

Perhaps the most consequential impact for women and their families resulting from this work is less obvious. A once‐in‐one‐hundred‐year pandemic risked a fear‐based, reactionary response to this aspect of the human experience. At a time of global uncertainty and personal insecurity, the availability of contemporaneous, evidenced‐based advice provided clinicians, and pregnant women, with reassurance that most, if not all, of the usual pregnancy, birthing and early parenting experiences did not have to change because of SARS‐CoV‐2.

## Open access

Open access publishing facilitated by Monash University, as part of the Wiley – Monash University agreement via the Council of Australian University Librarians.

## Competing interests

No relevant disclosures.

## Provenance

Not commissioned; externally peer reviewed.
